# Familial 22q11.2 Duplication/Deletion Syndrome: A Testament to the Long‐Standing Clinical Utility of FISH

**DOI:** 10.1002/ccr3.71483

**Published:** 2025-11-14

**Authors:** Laura M. Bryant, Rose Hokanson, Scott E. Hickey, Bianca Zapanta, Jesse Hunter, Shalini C. Reshmi

**Affiliations:** ^1^ The Steve and Cindy Rasmussen Institute for Genomic Medicine Nationwide Children's Hospital Columbus Ohio USA; ^2^ Department of Pediatrics, Section of Genetic and Genomic Medicine Nationwide Children's Hospital Columbus Ohio USA; ^3^ Department of Pediatrics The Ohio State University Columbus Ohio USA; ^4^ The Ohio State University Columbus Ohio USA

**Keywords:** 22q deletion, 22q duplication, chromosomal microarray, FISH

## Abstract

While the standard diagnostic test for suspected 22q copy number disorders is by chromosomal microarray, this case highlights the importance of utilizing FISH to localize allelic copy number when there is a family history of deletion/duplication syndrome for accurate recurrence risk assessment.

## 1 Introduction

The chromosome 22q11.2 band region is associated with multiple genomic disorders resulting from low copy repeats (LCRs) clustered around chromosome‐specific segmental duplications that undergo nonallelic homologous recombination during meiosis [1]. These exchanges can give rise to both gains and losses of genetic material. The 22q11.2 microdeletion syndrome (MIM# 188400) is also referred to as DiGeorge syndrome, velocardiofacial syndrome, Shprintzen syndrome and conotruncal anomaly face syndrome depending upon clinical presentation [2]. There is wide phenotypic variability across these disorders, including: hypocalcemia (due to parathyroid hypoplasia), facial dysmorphism, congenital heart disease (particularly conotruncal malformations including ventricular septal defect, tetralogy of Fallot, interrupted aortic arch type B, and truncus arteriosus), palatal defects, immune deficiency (thymic hypoplasia), developmental delay, autism, and/or ADHD [2, 3, 4, 5, 6]. While approximately 95% of 22q microdeletions are de novo, most 22q microduplications (MIM# 608363) are frequently inherited from an unaffected or mildly affected parent [2, 7] as phenotypic findings may be subclinical (or asymptomatic). To our knowledge, this is the first report of familial segregation demonstrating both 22q11.2 microduplication and microdeletion syndromes within a single pedigree.

## 2 Case History

**FIGURE 1:**
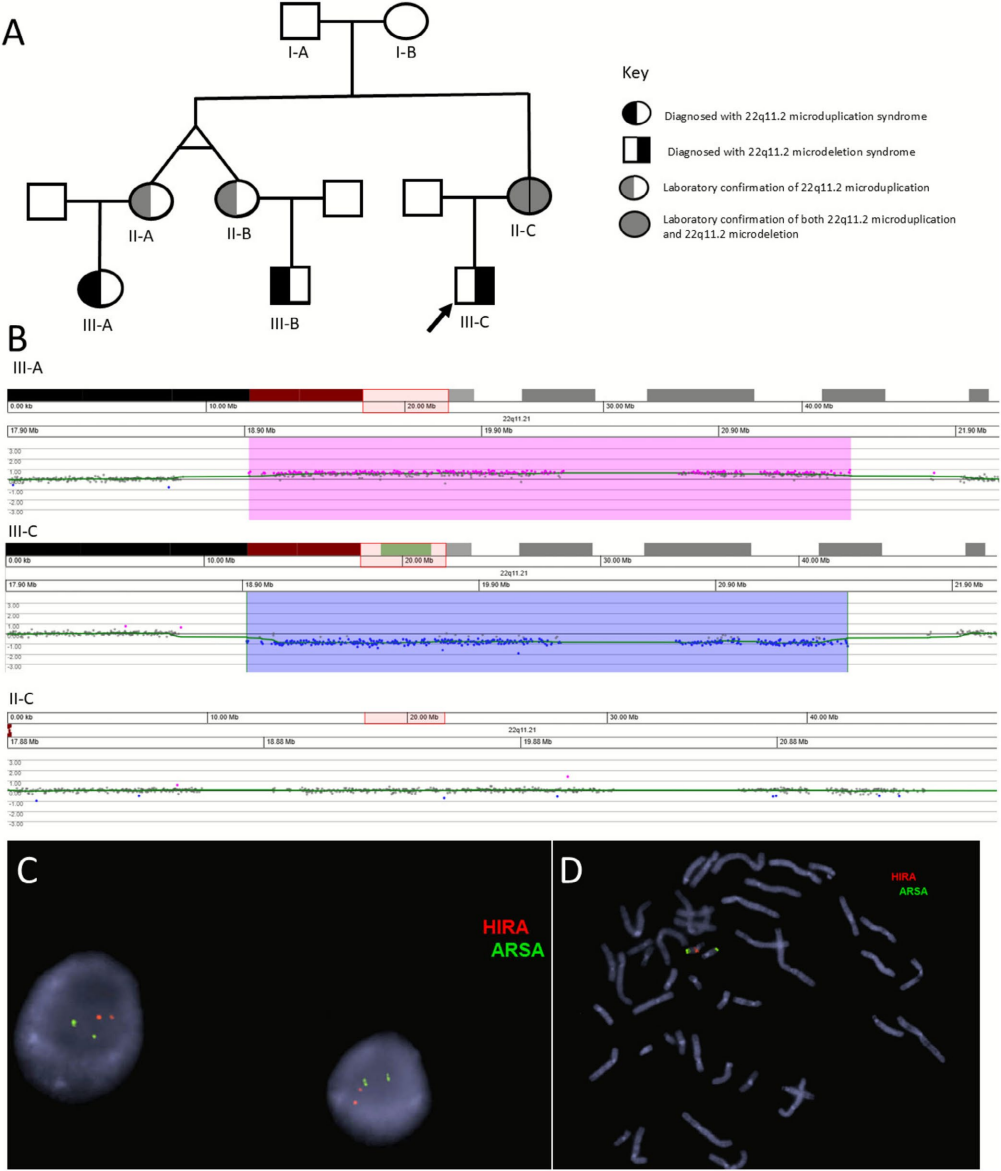
(A) Family pedigree. Left fill‐in indicates 22q11.2 microduplication and right fill‐in indicates 22q11.2 microdeletion. Black indicates clinical diagnosis; gray indicates laboratory confirmation of the duplication or deletion in an asymptomatic or mildly affected individual without a clinical diagnosis. Proband indicated by arrow. (B) CGH microarray findings from three family members. III‐A demonstrates the 22q11.2 microduplication while III‐C shows a 22q11.2 microdeletion and II‐C shows a normal copy state. (C) Interphase FISH showing two copies of the *HIRA* locus (22q11.2 region) and *ARSA* (proximal to chromosome 22 centromere). (D) Metaphase FISH demonstrating a single signal for the *HIRA* locus and lack of signal for the locus on the other homolog (indicated by the presence of the *ARSA* control probe).

A 7‐month‐old male (Figure 1, III‐C) presented with mild delay in gross and fine motor skills, a small patent foramen ovale, subtle dysmorphic features, including epicanthal folds and a thin upper lip, and a strong family history of 22q11.2 microduplication syndrome (Figure 1, III‐A and III‐B). Notably, two maternal cousins had previously been diagnosed with 22q11.2 microduplication syndrome and were severely affected, with congenital structural defects requiring surgical intervention. At that visit, the two maternal aunts (presumed to be monozygotic twins) were reported to also have the 22q11.2 duplication but were reported to be either subclinical or asymptomatic (information from reported family history alone).

## 3 Methods

DNA extracted from peripheral blood was utilized for microarray analysis. DNA extraction was performed using commercial methods on a Chemagic 360 (Perkin Elmer; Shelton, CT). The DNA was run on a custom‐designed Agilent 180k CGH+SNP array with ~120,000 probes spaced on average one probe every 25 kb throughout the genome with higher density in regions known to have clinical significance. Data were analyzed using Agilent CytoGenomics version 5.2 (Santa Clara, CA). The analysis was based on the human reference genome build (GRCh37/hg19). Fluorescence in situ hybridization was performed on peripheral blood using direct‐labeled probes harboring the DiGeorge critical gene, *HIRA*, within the 22q11.2 region along with a control gene, *ARSA*, that is distal to the DiGeorge region at 22q13.3 (Abbott Molecular; Abbott Park, IL).

## 4 Conclusions and Results

Microarray analysis of the proband revealed the common, 2.54 Mb deletion within chromosome band 22q11.2, spanning low copy repeat breakpoints A through D (Figure 1B). Echocardiogram revealed a mild physiologic peripheral branch stenosis, but physical examination did not detect any physical symptoms associated with 22q11.2 microdeletion syndrome. Subsequent SNP microarray was performed on the proband's mother (II‐C) and demonstrated a normal, two‐copy state for the 22q11.2 region (Figure 1B). Given the strong family history of 22q11.2 microduplication, FISH was performed on peripheral blood from the proband's mother. Interphase analysis revealed two signals for the *HIRA* probe; however, metaphase cells revealed one large signal from the *HIRA* probe on a single chromosome 22 homolog, and no signal for the *HIRA* locus on the other chromosome 22 homolog (Figure 1C,D). Taken together, these results were consistent with the previous array finding of a normal copy number state, but demonstrated an apparently balanced rearrangement wherein one chromosome homolog harbored a 22q11.2 microduplication and the other chromosome homolog was deleted for the 22q11.2 locus.

The proband had clinic follow‐up at 2 years, 3 months of age and knows 50+ words and is working on two‐word phrases. He had been evaluated by Urology for retractile testes, but no intervention was needed. On ophthalmologic evaluation, he was found to have bilateral esophoria and mild myopia and astigmatism. Growth is thus far within the normal range. His mother has no major health problems and denies a history of learning concerns.

A maternal aunt was reported to have 22q11.2 duplication syndrome and had a 504 plan for reading in school and a history of supraventricular tachycardia. Her daughter (maternal first cousin), also with 22q11.2 duplication syndrome, was found shortly after birth to have severe Ebstein's anomaly, moderate to severe tricuspid valve regurgitation, and anterior muscular ventricular septal defect. This cousin underwent tricuspid valve repair and reduction atrioplasty complicated by severe right ventricular dysfunction with low cardiac output that led to the need for an emergent Glenn procedure. She was last evaluated at 2 years, 9 months without apparent developmental delays.

## 5 Discussion

This case was unusual in that the proband studied presented with a strong family history of 22q11.2 microduplication, but no reports of 22q11.2 loss. Although the proband had a very mild presentation of 22q11.2 microdeletion syndrome, his cousins (III‐A and III‐B) both presented with symptoms that were more severe than those typically described for 22q11.2 microduplication syndrome. It is unclear if additional genetic testing was carried out for these individuals. While there is significant overlap between 22q11.2 microduplication syndrome and 22q11.2 microdeletion syndrome, it is generally common for the microdeletion to present with more severe medical complications. Indeed, the aunts of the proband (II‐A and II‐B) were mildly affected/unaffected, consistent with the wide intrafamilial phenotypic variability that has been previously reported [7].

It is possible, but less likely, that the deletion in our index case occurred de novo, despite a positive history for 22q11.2 microduplication. Based solely on the microarray finding of a normal copy number for the 22q11.2 region in the proband's mother, the recurrence risk for having another affected child would be low, considering the possibility of gonadal mosaicism. However, follow‐up FISH studies demonstrated that the mother of the affected individual was an asymptomatic, balanced carrier of both the 22q11.2 microduplication and the 22q11.2 microdeletion. This finding has a significant effect on counseling for recurrence risk, as all subsequent offspring would be predicted to harbor a 22q microdeletion or 22q microduplication. Although the simultaneous presence of both a deletion and duplication at this locus in a single individual is a rare phenomenon—previously reported only once [8]—our findings highlight the value of FISH studies as an important complement to microarray testing. This approach enables identification of rare cases involving concurrent deletion and duplication rearrangements in a carrier parent.

## Author Contributions

**Laura M. Bryant:** conceptualization, formal analysis, writing – original draft. **Rose Hokanson:** data curation, writing – review and editing. **Scott E. Hickey:** data curation, formal analysis, writing – review and editing. **Bianca Zapanta:** data curation, formal analysis, writing – review and editing. **Jesse Hunter:** formal analysis, writing – review and editing. **Shalini C. Reshmi:** conceptualization, formal analysis, writing – original draft.

## Consent

Written consent for publication of clinical data was obtained by the department of clinical genetics from the proband's mother.

## Conflicts of Interest

The authors declare no conflicts of interest.

## Data Availability

Data sharing not applicable to this article. All relevant data is included in the article and further information is not available due to privacy considerations.

## References

[1] B. S. Emanuel , “Molecular Mechanisms and Diagnosis of Chromosome 22q11.2 Rearrangements,” Developmental Disabilities Research Reviews 14, no. 1 (2008): 11–18, 10.1002/ddrr.3. 18636632 PMC2810965

[2] D. M. McDonald‐McGinn , H. S. Hain , B. S. Emanuel , and E. H. Zackai , “22q11.2 Deletion Syndrome,” in GeneReviews, ed. M. P. Adam , J. Feldman , G. M. Mirzaa , R. A. Pagon , S. E. Wallace , and A. Amemiya (University of Washington, 1993), http://www.ncbi.nlm.nih.gov/books/NBK1523/. 20301696

[3] I. M. Campbell , S. E. Sheppard , T. B. Crowley , et al., “What Is New With 22q? An Update From the 22q and You Center at the Children's Hospital of Philadelphia,” American Journal of Medical Genetics, Part A 176, no. 10 (2018): 2058–2069, 10.1002/ajmg.a.40637. 30380191 PMC6501214

[4] A. Cirillo , M. Lioncino , A. Maratea , et al., “Clinical Manifestations of 22q11.2 Deletion Syndrome,” Heart Failure Clinics 18, no. 1 (2022): 155–164, 10.1016/j.hfc.2021.07.009. 34776076

[5] D. M. McDonald‐McGinn , K. E. Sullivan , B. Marino , et al., “22q11.2 Deletion Syndrome,” Nature Reviews Disease Primers 1 (2015): 15071, 10.1038/nrdp.2015.71. PMC4900471 27189754

[6] A. Tomita‐Mitchell , D. K. Mahnke , J. M. Larson , et al., “Multiplexed Quantitative Real‐Time PCR to Detect 22q11.2 Deletion in Patients With Congenital Heart Disease,” Physiological Genomics 42A, no. 1 (2010): 52–60, 10.1152/physiolgenomics.00073.2010. 20551144 PMC2957771

[7] L. E. Bartik , S. S. Hughes , M. Tracy , et al., “22q11.2 Duplications: Expanding the Clinical Presentation,” American Journal of Medical Genetics. Part A 188, no. 3 (2022): 779–787, 10.1002/ajmg.a.62577. 34845825

[8] N. Carelle‐Calmels , P. Saugier‐Veber , F. Girard‐Lemaire , et al., “Genetic Compensation in a Human Genomic Disorder,” New England Journal of Medicine 360, no. 12 (2009): 1211–1216, 10.1056/NEJMoa0806544. 19297573

